# Dietary Transitions and the Rising Global Burden of Chronic Kidney Disease: Insights from Nutritional Epidemiology

**DOI:** 10.3390/nu18060911

**Published:** 2026-03-13

**Authors:** Fabián Vásquez, Caterina Tiscornia, Valeria Aicardi, Sofía Vásquez

**Affiliations:** 1 Escuela de Nutrición y Dietética, Universidad Finis Terrae, Santiago 7501015, Chile; fvasquez@uft.cl; 2 Unidad de Diálisis, Clínica Indisa, Santiago 7530104, Chile; valeria.aicardi@gmail.com; 3 Escuela de Medicina, Universidad de Chile, Santiago 8380000, Chile; sofia.vasquez.o@ug.uchile.cl

**Keywords:** chronic kidney disease, dietary transitions, nutritional epidemiology, ultra-processed foods, diet quality, gut–kidney axis, cardio-kidney-metabolic syndrome, non-communicable disease

## Abstract

**Background/Objectives:** Chronic kidney disease (CKD) is one of the fastest-growing non-communicable diseases globally, with a disproportionate burden in populations undergoing rapid dietary and epidemiological transitions. Beyond traditional clinical risk factors, increasing evidence from nutritional epidemiology suggests that contemporary dietary environments may play a significant role in shaping CKD risk, complications, and progression. This narrative review examines CKD as a potential unintended consequence of global dietary transitions, with particular emphasis on ultra-processed foods and overall diet quality. **Methods:** A structured narrative review was conducted using evidence from prospective cohort studies, systematic reviews, meta-analyses, and mechanistic research. Findings were synthesized within a population-health framework integrating dietary patterns, food processing classification, and biologically plausible pathways relevant to kidney health. **Results:** Healthier dietary patterns, including Mediterranean, DASH, and plant-forward diets, are consistently associated with lower risk of incident CKD, slower kidney function decline, and reduced mortality. In contrast, Western dietary patterns characterized by high intake of ultra-processed foods are linked to increased CKD risk and adverse cardiometabolic outcomes. Beyond mediators such as hypertension, diabetes, and obesity, emerging mechanisms include dietary acid load, gut dysbiosis and uremic toxin production, sodium density, and exposure to highly bioavailable phosphate additives. **Conclusions:** Dietary transitions toward ultra-processed, low-fiber, sodium- and additive-rich food environments may contribute to the growing global burden of CKD through interconnected metabolic, inflammatory, and gut–kidney pathways. Improving diet quality and addressing food processing at the population level represent promising opportunities for CKD prevention and risk reduction.

## 1. Introduction

CKD has emerged as one of the fastest-growing contributors to global morbidity and mortality, representing a major and often underrecognized public health challenge. Recent estimates from the GBD Study indicate that CKD affects more than 850 million people worldwide and ranks among the leading causes of years of life lost, with a sustained increase in prevalence over the past three decades [1,2]. Unlike many other NCDs, CKD has shown limited progress in prevention and early detection, contributing to substantial healthcare costs and premature mortality.

CKD is closely intertwined with CVD and metabolic disorders, particularly hypertension, type 2 diabetes mellitus, and obesity, which together account for most CKD cases globally [3,4]. Reduced kidney function and albuminuria are powerful independent predictors of cardiovascular events and all-cause mortality, reinforcing the concept of CKD as a systemic condition rather than an isolated renal disorder [5]. This bidirectional relationship amplifies disease burden and complicates prevention strategies, especially in aging populations.

Importantly, the burden of CKD is not evenly distributed. LMICs bear a disproportionate share of CKD-related morbidity and mortality, driven by rapid epidemiological transitions, limited access to early diagnosis, and constrained health system capacity [2,6]. Evidence on CKD underdiagnosis in LMICs derives primarily from GBD modeling studies and population-based surveys, which consistently show lower detection rates and delayed presentation compared with high-income settings [2,6]. In addition, reports from regional health system analyses and hospital-based registries indicate that CKD is frequently identified only at advanced stages, when therapeutic options are limited and costly [6]. Importantly, the burden of CKD is not evenly distributed. LMICs bear a disproportionate share of CKD-related morbidity and mortality, driven by rapid epidemiological transitions, limited access to early diagnosis, and constrained health system capacity. In these settings, CKD frequently remains undiagnosed until advanced stages, when therapeutic options are limited and costly. Beyond health-system constraints, epidemiological evidence indicates that LMICs undergoing rapid nutrition transitions are increasingly exposed to structural dietary shifts, including the expansion of UPF availability and sales linked to food-system transformations, urbanization, and retail market penetration. Multi-country analyses documenting global UPF sales show substantial growth across transitioning economies, with wide regional variation and faster increases in many middle-income settings. Authoritative reports focused on children’s food environments similarly describe rapidly rising UPF intake in LMICs in parallel with worsening cardiometabolic risk profiles [2,6].

The concept of the nutrition transition describes large-scale shifts in dietary patterns that accompany economic development, urbanization, and globalization, moving from traditional diets rich in minimally processed foods toward industrialized dietary patterns dominated by UPFs [7,8]. While the nutrition transition refers specifically to shifts in dietary patterns and food systems associated with economic development and globalization, it is conceptually distinct from the epidemiological transition, which describes broader changes in disease patterns from infectious to chronic non-communicable diseases over time. The nutrition transition is characterized by increased consumption of energy-dense, nutrient-poor products, high intakes of sodium, added sugars, and refined fats, and a parallel decline in dietary fiber, fruits, vegetables, legumes, and whole grains [9,10].

According to the NOVA food classification system [11,12]:

- Unprocessed or minimally processed foods include natural edible parts of plants and animals (e.g., fresh fruits, vegetables, grains, milk, meat) that have undergone minimal alterations such as cleaning, grinding, refrigeration, or pasteurization.
- Processed foods are products made by adding salt, sugar, oil, or other culinary ingredients to unprocessed foods to enhance durability or palatability (e.g., canned vegetables, cheese, freshly baked bread).
- Ultra-processed foods (NOVA category 4) are industrial formulations made mostly or entirely from substances extracted from foods or synthesized in laboratories, typically containing little or no intact whole food, and including additives such as flavor enhancers, emulsifiers, preservatives, and colorings. Examples include packaged snacks, sugary beverages, processed meats, instant noodles, and industrial baked goods.

UPFs, as classified by the NOVA system, have become a dominant source of dietary energy in many countries, accounting for more than 50% of total caloric intake in some high-income populations and rising rapidly in LMICs [11,12]. These products typically contain high levels of sodium and phosphorus-based additives, low potassium-to-sodium ratios, and limited bioactive compounds, features that are increasingly recognized as relevant to renal and cardiometabolic health [13].

Strikingly, the global expansion of UPFs consumption has occurred in parallel with the rising incidence and prevalence of CKD. While this temporal coincidence does not establish causality, it raises important questions about the role of dietary environments in shaping kidney health at the population level [14]. The convergence of dietary transitions with epidemics of hypertension, diabetes, and obesity suggests that modern food systems may contribute both indirectly and directly to CKD risk and progression, supporting the central hypothesis of this review. Although most available evidence derives from prospective cohort studies, the possibility of reverse causality cannot be entirely excluded, as individuals with early or subclinical CKD may have modified their dietary habits prior to formal diagnosis.

Growing evidence from nutritional epidemiology supports diet as a key modifiable determinant of CKD onset and progression. Prospective cohort studies and meta-analyses consistently show that healthier dietary patterns such as the Mediterranean and DASH diets are associated with lower risk of incident CKD, slower decline in estimated glomerular filtration rate, and reduced mortality among individuals with established kidney disease [15,16,17]. Conversely, Western dietary patterns characterized by high intakes of red and processed meats, sodium, and ultra-processed foods have been linked to higher CKD risk and adverse renal outcomes [18,19].

In this review, diet quality refers to the overall alignment of habitual dietary intake with evidence-based dietary patterns associated with cardiometabolic and renal protection. It is typically operationalized using validated indices such as HEI, Mediterranean Diet Scores, or DASH scores, which integrate multiple food groups and nutrient domains rather than single nutrients. Higher diet quality generally reflects greater consumption of fruits, vegetables, whole grains, legumes, and unsaturated fats, and lower intake of sodium, refined carbohydrates, red and processed meats, and ultra-processed foods.

Low-quality dietary patterns are commonly characterized by high energy density, high sodium density, low dietary fiber intake, low fruit and vegetable consumption, and a high proportion of total energy derived from ultra-processed foods. These features serve as practical surrogates of poor diet quality in epidemiologic studies. The term lower-quality fat profile refers to dietary patterns in which saturated and industrial trans fats predominate over mono- and polyunsaturated fats. Such profiles are typically observed in diets rich in processed meats, fried foods, and commercially baked products, and have been associated with endothelial dysfunction and cardiometabolic risk.

From a public health perspective, population-based dietary strategies offer substantial advantages over individual-level nutritional interventions. Small shifts in average dietary sodium, fiber, or UPFs intake across entire populations can yield large aggregate reductions in cardiometabolic and renal disease burden [20]. However, despite the growing epidemiological evidence, a significant gap persists between nutrition science and food policy implementation. Current dietary guidelines and regulatory frameworks often fail to address the structural drivers of unhealthy diets, particularly the widespread availability and marketing of ultra-processed foods [21].

Bridging this gap is especially critical for CKD, a condition that remains largely absent from global NCD prevention agendas despite its strong dietary determinants and profound societal impact. Integrating nutritional epidemiology with clinical nephrology and public health policy is therefore essential to advance effective, equitable prevention strategies.

**Figure 1:**
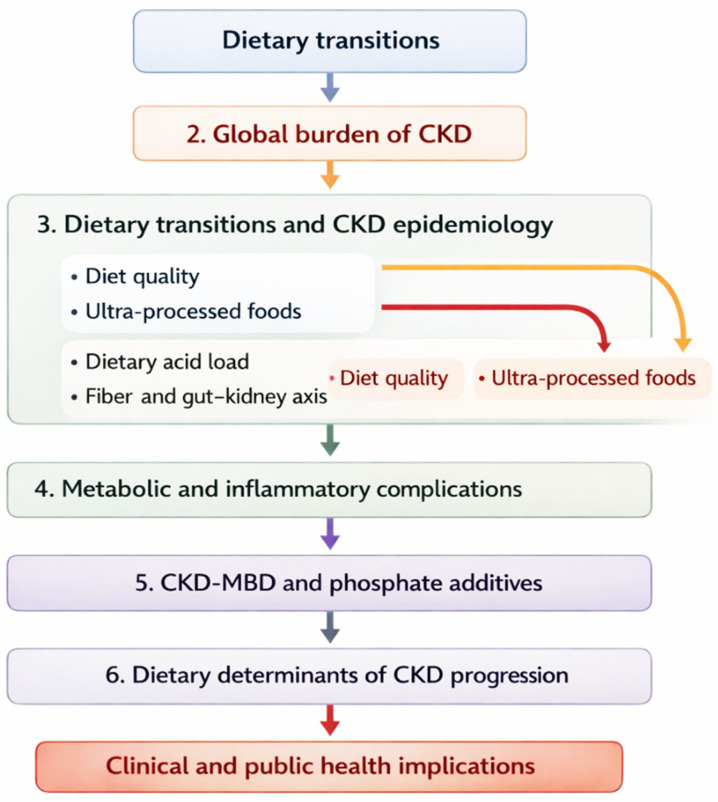
Structural overview of the review.

The aim of this narrative review is to examine chronic kidney disease as a potential unintended consequence of global dietary transitions. By integrating evidence from nutritional epidemiology, clinical research, and population health studies, we seek to elucidate the pathways linking modern dietary patterns to CKD risk and progression, and to discuss the clinical and public health implications of addressing diet as a central lever for CKD prevention. To facilitate conceptual navigation of the review and provide readers with a structured overview of the main themes discussed across sections, Figure 1 presents the overall organization of the article.

This schematic diagram illustrates the conceptual organization of the review and the progression of topics across sections. The article begins by situating CKD within the broader context of global dietary transitions and their potential implications for kidney health. It then examines the epidemiological links between modern dietary environments and CKD risk, with particular attention to diet quality, ultra-processed food consumption, dietary acid load, and fiber-related gut–kidney interactions. Subsequent sections address the downstream metabolic and inflammatory complications associated with these dietary exposures, followed by the role of phosphate additives and CKD-mineral and bone disorder. The review then discusses dietary determinants of CKD progression and secondary prevention strategies. The final section synthesizes the clinical and public health implications of these findings, highlighting the relevance of dietary patterns and food-system environments for CKD prevention and management.

This structural overview is intended to guide readers through the conceptual architecture of the review and to clarify how the different thematic sections contribute to understanding the role of dietary transitions in CKD development and progression.

## 2. Materials and Methods

This narrative review was grounded in methodological principles commonly used in nutritional epidemiology and population health research, including the evaluation of dietary patterns rather than isolated nutrients, consideration of food-processing classifications (e.g., NOVA), integration of epidemiological and mechanistic evidence, and interpretation of diet–disease relationships within population-level contexts. Although the review was not designed as a formal systematic review, it followed key elements of structured evidence synthesis, including a predefined search strategy, explicit inclusion and exclusion criteria, and a theory-driven conceptual framework linking dietary transitions with CKD risk and progression. This approach aimed to maintain methodological rigor while allowing the flexibility needed to explore complex and multidimensional dietary exposures and health outcomes.

The literature search was conducted between 1 September 2025 and 31 January 2026 across several major databases, including PubMed/MEDLINE, Scopus, Web of Science, and ScienceDirect, with a predefined final cut-off date of 31 January 2026. No rolling updates were performed after this date. To ensure thorough coverage, the search was supplemented with university library resources and authoritative reference texts in nephrology, nutrition, and public health. The strategy combined MeSH terms and free-text keywords related to chronic kidney disease, dietary transitions, and cardiometabolic outcomes. Boolean operators were used to reflect the multidimensional nature of dietary patterns and their effects on kidney and overall health. Search terms included combinations of “chronic kidney disease”, “CKD”, “kidney function decline”, and “albuminuria” with “nutrition transition”, “dietary patterns”, “Mediterranean diet”, “DASH diet”, “plant-based diet”, as well as “ultra-processed foods”, “food processing”, “NOVA classification”, “sodium intake”, “phosphate additives”, “dietary phosphorus”, and further with “cardiovascular disease”, “inflammation”, “obesity”, and “insulin resistance”. Key article references and “cited by” lists were also manually reviewed to help ensure no relevant studies were missed. Database filters were applied to restrict results to human studies and adult populations where available; however, no restrictions were imposed on study design at the search stage, and eligibility criteria were subsequently applied during screening.

Inclusion criteria were established in advance. Studies were considered if they were published up to the date indicated above, reported original research, narrative or systematic reviews, or meta-analyses; involved adult populations (with mechanistic or experimental studies included when necessary to support biological relevance); and examined dietary patterns, ultra-processed food intake, sodium or phosphate exposure, or broader nutrition transitions in connection with CKD development, progression, or related cardiometabolic outcomes. Articles in English or Spanish were eligible. Studies were excluded if full-text access was unavailable, if the record was a duplicate, if the focus was solely on acute kidney injury or on non-dietary causes of kidney disease, or if the source lacked peer review or methodological clarity.

After removing duplicates, titles and abstracts were screened for relevance, and full-text reviews were conducted to confirm inclusion. Selection was based on conceptual fit, methodological rigor, and relevance to population-level kidney health rather than strictly clinical outcomes. Data were extracted using a standardized form, covering publication year and location, study design (e.g., prospective cohort, cross-sectional, RCT, review, meta-analysis), population type (general population, individuals with CKD, or high-risk groups), how dietary exposure was assessed (e.g., pattern-based, nutrient-specific, food processing level), and the main renal and cardiometabolic outcomes. Key findings, limitations, confounding adjustment strategies, and effect estimates were also recorded when available. Titles and abstracts were screened independently by two reviewers to assess eligibility. Full-text articles were evaluated using the predefined inclusion and exclusion criteria. Disagreements were resolved through consensus discussion; unresolved cases were adjudicated by a third reviewer to ensure methodological consistency.

The evidence was synthesized using a conceptual framework that addressed global CKD trends, cardiometabolic comorbidities, dietary shifts at the population level, complications related to kidney disease (cardiovascular, metabolic, inflammatory, mineral-bone), and the dietary factors involved in CKD progression, especially for secondary prevention. Studies were organized by epidemiological level—population-based, clinical CKD groups, and mechanistic research. A narrative synthesis was carried out with emphasis on consistency of associations, methodological quality, dose–response relationships, and biological plausibility. Greater interpretive value was placed on prospective studies, large cohorts, and meta-analyses, while cross-sectional and mechanistic studies were used to support and contextualize findings.

Although no formal quantitative risk-of-bias tools were applied, study quality was assessed qualitatively, considering aspects like study design, dietary exposure accuracy, outcome definitions, and confounding control. A formal risk-of-bias tool (e.g., ROBINS-I or AMSTAR-2) was not applied because the objective of this narrative review was to provide a theory-informed integrative synthesis across heterogeneous study designs rather than to conduct a pooled quantitative analysis. Any disagreements during study selection or interpretation were resolved by consensus among the authors.

## 3. Global Burden of Chronic Kidney Disease

### 3.1. Dietary Transitions and CKD-Related Complications

CVD remains the leading cause of morbidity and mortality across the CKD spectrum, and dietary transitions toward ultra-processed, sodium-dense, and energy-dense food environments likely amplify this risk through both indirect cardiometabolic pathways and direct cardiorenal mechanisms [1,22]. From a nutritional epidemiology perspective, the most consistent signals relate to overall diet quality (dietary patterns), sodium and sodium-to-potassium balance, fat quality, UPFs, each of which have been altered substantially by global dietary transitions. From a nutritional epidemiology perspective, the most consistent signals relate to overall diet quality (dietary patterns), sodium and sodium-to-potassium balance, fat quality, and ultra-processed food exposure. These components have all undergone substantial modification as a consequence of global dietary transitions.

### 3.2. Low Diet Quality and CVD Risk in CKD

Observational evidence and synthesis work increasingly support that healthier dietary pattern (e.g., Mediterranean-like, DASH-like, plant-forward patterns) are associated with lower cardiometabolic risk profiles and improved renal outcomes, whereas Western/UPF-dominant patterns cluster with higher BP, dyslipidemia, obesity, and diabetes, all key mediators of CVD in CKD [23,24]. Importantly, CKD magnifies vulnerability to dietary exposures because vascular dysfunction, oxidative stress, and uremia-related inflammation lower the threshold at which adverse dietary profiles translate into clinical outcomes [25]. Recent umbrella-level syntheses of dietary patterns and kidney health also support clinically relevant effects for patterns that reduce proteinuria and improve risk factors closely linked to CVD (BP control, metabolic regulation) [23].

### 3.3. Role of Sodium: Beyond BP

Sodium density rises markedly with industrial processing, making dietary transitions a primary driver of high sodium intake at the population level. In CKD, sodium excess is strongly tied to hypertension, volume expansion, and proteinuria, and these pathways jointly increase CVD risk and accelerate renal functional decline [26,27]. Evidence syntheses in broader populations continue to support an adverse association between higher sodium intake and cardiovascular outcomes, while in CKD the clinical and public health challenge is achieving effective sodium reduction in a food environment where most sodium originates from processed/ultra-processed products rather than discretionary salt [26]. In addition, emerging work highlights the relevance of the dietary Na/K ratio as a risk marker for cardiovascular events and mortality, biologically plausible given countervailing effects of potassium-rich minimally processed diets on vascular tone and natriuresis [28]. In practice, Na/K ratio offers a useful population-facing indicator that aligns with dietary transition dynamics (UPFs increase Na while reducing K from fresh foods).

### 3.4. Fats, UPFs, and the “Cardio–Kidney–Metabolic” Interface

Dietary transitions often entail increased intake of refined fats and lower-quality fat profiles alongside high glycemic load foods and low fiber. UPFs represent a particularly informative exposure because they capture multiple co-occurring features—high sodium density, refined fats, added sugars, low fiber content, and various industrial additives-while also reflecting the structural shift from home-prepared to industrialized food systems [22,29]. Large evidence syntheses conducted predominantly in general population cohorts consistently associate higher UPF consumption with adverse cardiometabolic outcomes, including CVD. A recent 2024–2025 dose–response meta-analysis of prospective studies further confirmed that greater UPF intake is associated with increased risk of incident CVD and cardiovascular mortality, strengthening the epidemiologic signal linking industrial food environments to vascular risk. Mechanistic reviews in non-CKD populations emphasize systemic inflammation, endothelial dysfunction, insulin resistance, oxidative stress, and gut dysbiosis as plausible biological pathways [22,29]. In individuals with CKD, direct mechanistic data remain more limited; however, these pathways are biologically plausible and likely additive to uremia-related inflammation and oxidative stress, potentially amplifying cardiovascular vulnerability in this high-risk group. A recent clinical-scientific statement from cardiovascular organizations also summarizes the epidemiologic evidence linking UPFs to cardiometabolic disease and highlights the relevance of this exposure for prevention and policy discussions [30]. Emerging cardio-kidney-metabolic frameworks further situate UPFs within an interconnected pathway network linking obesity, diabetes, CVD, and kidney disease, supporting an integrated prevention lens rather than siloed disease models [31].

### 3.5. Implications Under Dietary Transition

From a population health viewpoint, CVD risk in CKD is increasingly shaped by food environments: sodium-rich ultra-processed staples, aggressive marketing, and affordability gradients that concentrate exposure in disadvantaged groups. This aligns with the growing interest in policy levers (reformulation, front-of-pack labeling, marketing restrictions, fiscal policies) as complements to individual counseling, especially when CKD patients attempt sodium restriction within a high-sodium food supply [29,30,31]. In secondary prevention (established CKD), dietary strategies that simultaneously reduce sodium density, improve fat quality, and shift consumption away from UPFs may yield “multi-risk-factor” benefits (BP, proteinuria, metabolic control), with plausible downstream effects on CVD event rates.

## 4. Dietary Transitions and the Epidemiology of CKD

The contemporary epidemiology of CKD is best understood within the framework of the nutrition transition: the progressive replacement of traditional preparations and home-cooked meals by ready-to-consumer products with higher energy density and greater proportions of sodium, added sugars, unhealthy fats, and food additives. This transition aligns with a “Western” dietary pattern and with the rapid expansion of UPF markets globally. For example, in countries such as Mexico and Brazil, national dietary surveys have documented a marked increase in the contribution of UPFs to total energy intake over the past two decades, accompanied by rising prevalence of obesity, diabetes, and hypertension—key upstream determinants of CKD. This pattern has been similarly observed across several low- and middle-income countries undergoing rapid urbanization, illustrating how structural changes in food systems may shape renal risk at the population level [32].

From both pathophysiological and population perspectives, the Western dietary pattern is characterized by high intake of animal protein (particularly red and processed meats), low consumption of fruits, vegetables, and dietary fiber, and a high burden of industrially processed foods. Collectively, these components are associated with an increased risk of CKD through plausible and convergent mechanisms, including glomerular hemodynamic changes (hyperfiltration), increased endogenous acid load with a greater requirement for ammoniagenesis, and the coexistence of obesity, hypertension, and diabetes, which mediate a substantial proportion of renal risk [33].

Current evidence synthesized at the level of dietary patterns shows consistent associations. In a systematic review and meta-analysis of observational studies (17 studies; approximately 150,000 participants), higher adherence to a “healthy” dietary pattern was associated with a lower likelihood of CKD (OR ≈ 0.69), whereas higher adherence to a “Western” dietary pattern was associated with a higher likelihood of CKD (OR ≈ 1.86). This contrast supports the notion that population-level dietary shifts toward Western patterns represent a relevant component in explaining trends in renal risk, particularly through their interaction with metabolic determinants [34].

### 4.1. Diet Quality and CKD Risk

In renal nutritional epidemiology, a priori indices (e.g., HEI, Mediterranean-style scores, and DASH scores) are commonly used to quantify overall diet quality and facilitate comparisons across populations. Although these indices differ in component weighting, scoring algorithms, and dietary assessment methods across studies, they were interpreted in this review as conceptually comparable markers of adherence to high-quality dietary patterns rich in plant-based foods and low in sodium and ultra-processed products. The included studies did not apply harmonized scoring systems; rather, associations were synthesized qualitatively, acknowledging methodological heterogeneity in exposure assessment [35].

From a “dietary transition” perspective, traditional diets, more strongly centered on fresh foods and home-based culinary preparations, tend to preserve higher nutrient density and lower exposure to UPFs. In contrast, Western dietary patterns incorporate greater amounts of processed meats, refined grains, sweets, and fast foods. In quantitative syntheses of dietary patterns, healthy patterns (characterized by high intakes of fruits, vegetables, whole grains, and other protective components) are associated with lower CKD risk, whereas Western patterns are consistently associated with higher CKD risk [34].

Overall, the evidence converges on the notion that higher diet quality corresponds to lower renal risk, and that the core components of dietary quality—greater intakes of fruits, vegetables, and fiber; lower energy density; and reduced exposure to processed foods, are plausibly protective through mechanisms involving lower systemic inflammation, reduced metabolic dysregulation, and a more favorable hemodynamic and acid–base profile. Consistent with this framework, Western dietary patterns, characterized by high energy density, high animal protein intake, and low consumption of fruits and vegetables-are linked to increased susceptibility to renal injury and disease progression, particularly in contexts of reduced nephron reserve or coexisting obesity, hypertension, and diabetes [33,34].

### 4.2. Ultra-Processed Foods and Sodium Exposure

UPFs concentrate a high sodium density due to their industrial formulation and the extensive use of sodium-based additives serving preservative, antimicrobial, emulsifying, acidity-regulating, leavening, and palatability-enhancing functions. This “sodium load” derives not only from added salt but also from multiple sodium-containing compounds embedded in food matrices, which hinders consumers’ ability to accurately estimate true exposure and favors sustained intakes above recommended levels [36,37]. It should be noted that sodium exposure assessment differs across studies; while 24 h urinary sodium excretion is considered the reference method, most large epidemiologic cohorts rely on FFQs or dietary recalls, which are subject to measurement error, misclassification, and underestimation of true intake.

Beyond sodium density alone, ultra-processed foods also contain specific inorganic phosphate additives (e.g., sodium phosphate, phosphoric acid, pyrophosphates), potassium-based salts, nitrates/nitrites, and industrial emulsifiers such as carboxymethylcellulose and polysorbate-80. Inorganic phosphate additives are absorbed at rates approaching 90–100%, substantially higher than the bioavailability of organic phosphorus in natural foods, thereby increasing phosphate burden and stimulating fibroblast growth factor 23 (FGF23) secretion. Elevated FGF23 has been associated with left ventricular hypertrophy, endothelial dysfunction, and adverse cardiovascular outcomes in CKD. In parallel, sodium-based preservatives and flavor enhancers further amplify RAAS activation and intraglomerular hypertension. Certain emulsifiers may disrupt intestinal barrier integrity, promoting gut dysbiosis and increased production of uremic toxins such as indoxyl sulfate and p-cresyl sulfate. Additionally, high-temperature industrial processing generates advanced glycation end-products (AGEs), which activate the receptor for AGEs (RAGE), triggering NF-κB–mediated inflammatory signaling. Thus, the adverse renal impact of UPFs likely reflects the combined effect of additive load, altered food matrix, reduced fiber content, and processing-derived bioactive compounds rather than sodium excess alone [36,37,38,39].

From a nephrological perspective, chronic excess sodium intake is closely linked to arterial hypertension and poorer blood pressure control, a key causal axis in the initiation and progression of CKD. In patients with established CKD, high exposure to UPFs may further exacerbate uremic metabolic disturbances and complicate the management of comorbidities, reinforcing an adverse cardio-reno-metabolic trajectory [38,39].

The association with proteinuria and renal damage is supported by converging mechanisms. Sodium-induced hypertension increases intraglomerular pressure and promotes albuminuria and proteinuria; in parallel, UPF-rich dietary patterns typically coexist with higher energy density, poorer glycemic profiles, and dyslipidemia, contributing to endothelial dysfunction and chronic inflammation. In summary, sodium acts as a central, but not exclusive, component within the ultraprocessed dietary “package” that increases renal vulnerability [36,40].

In the ARIC study (median follow-up of 24 years), higher UPF consumption was associated with a 24% greater risk of incident CKD (highest vs. lowest quartile). Importantly, this association persisted after multivariable adjustment for blood pressure, diabetes status, body mass index, and other established CKD risk factors, suggesting that the relationship was not fully explained by traditional cardiometabolic intermediates. Additionally, substitution analyses indicated that replacing one daily serving of UPFs with minimally processed foods was associated with lower CKD risk, reinforcing both biological plausibility and the potential impact of dietary pattern modification [36].

At the level of aggregated evidence, a dose–response systematic review and meta-analysis (eight studies; six cohorts) showed that high UPF intake was associated with increased CKD risk (RR = 1.25; 95%CI: 1.09–1.42, *p* < 0.0001), and that each 10% increment in UPF consumption was associated with a higher risk (RR = 1.07; 95%CI: 1.04–1.10, *p* < 0.001), consistent with a dose–response relationship. This consistency is critical for positioning ultraprocessing as a relevant exposure within dietary transitions [41].

Finally, data from national dietary surveys and population-level estimates support the notion that processed and ultra-processed foods account for a substantial fraction of sodium intake. For instance, these products have been reported to contribute approximately 72% of total dietary sodium, in addition to excess intakes of simple sugars and phosphorus, thereby shaping a high-risk dietary environment for hypertension and CKD [37].

### 4.3. Protein Sources and Dietary Acid Load

DAL has emerged as a key integrative mechanism linking Western dietary patterns with the risk and progression of CKD. DAL reflects the balance between acid precursors, primarily derived from the metabolism of sulfur-containing amino acids abundant in animal proteins, and alkaline precursors, mainly potassium, magnesium, and calcium salts derived from fruits and vegetables. Diets with high DAL induce a state of subclinical metabolic acidosis, which may contribute to renal injury through multiple pathophysiological pathways, including increased tubular ammoniagenesis, activation of the renin–angiotensin–aldosterone system, and heightened oxidative stress and interstitial inflammation [42,43].

Epidemiological evidence consistently supports an association between higher DAL and increased CKD risk. Among older adults in the Korean Kangbuk Samsung cohort, higher quartiles of NEAP were associated with a progressive increase in the likelihood of CKD, whereas higher potassium intake showed an independent protective effect; notably, no significant association was observed with total protein intake [42]. These findings reinforce the notion that protein quality and dietary context, rather than protein quantity alone, are critical determinants of renal risk.

Concordant results have been reported in large Asian populations. A cross-sectional analysis of the China Health and Nutrition Survey (n = 7699) demonstrated that elevated NEAP values were independently associated with higher CKD prevalence, with a linear dose–response relationship persisting after adjustment for cardiometabolic factors. In contrast, the PRAL index showed less consistent associations. NEAP may offer greater sensitivity in epidemiologic settings because it primarily reflects the balance between protein-derived acid precursors and potassium intake, capturing the protein-potassium ratio that is central to diet-related acidogenic burden, whereas PRAL incorporates additional mineral components that may introduce variability depending on dietary assessment methods [44].

In clinical populations, DAL has also been linked to adverse metabolic and renal profiles. Among patients with CKD and type 2 diabetes mellitus, higher DAL scores were associated with dietary patterns rich in meats, eggs, and animal proteins and low in fruits, vegetables, and fiber; moreover, elevated DAL was linked to higher serum creatinine, poorer lipid profiles, and greater metabolic burden, particularly among women [45]. These data suggest that DAL may amplify renal vulnerability in the context of cardiometabolic multimorbidity [45].

By contrast, plant-based diets and predominantly plant-forward dietary patterns are consistently associated with lower DAL, higher intakes of potassium and fiber, and reduced exposure to highly bioavailable phosphorus and acidogenic proteins. Narrative and systematic reviews indicate that plant-based diets, including Mediterranean and DASH variants, not only reduce dietary acid load but are also associated with lower CKD incidence and a slower decline in kidney function, even in advanced stages of disease [45]. Mechanistically, the lower DAL of these diets mitigates metabolic acidosis, reduces proteinuria, and improves blood pressure control, thereby contributing to a more favorable metabolic environment for the preservation of renal function.

Taken together, the evidence suggests that shifting from acidogenic Western dietary patterns toward diets with a higher proportion of plant-derived foods represents a relevant and potentially cost-effective strategy for both primary and secondary prevention of CKD. This approach moves the focus away from isolated nutrient restriction toward comprehensive modulation of dietary patterns, positioning DAL as a key marker in the nutritional epidemiology of kidney disease [46].

### 4.4. Fiber Intake and Gut–Kidney Axis

#### 4.4.1. Reduction of Dietary Fiber in Modern Diets

Beyond dietary acid load, another critical pathway linking Western dietary patterns to CKD progression involves the interaction between dietary fiber intake, gut microbiota composition, and uremic toxin production. One of the most consistent nutritional features of Westernized dietary patterns is a marked reduction in total dietary fiber intake, driven by the displacement of whole grains, legumes, fruits, and vegetables by refined foods and UPFs. In patients with CKD, habitual dietary fiber intake is frequently well below recommended levels for the general population, with observational cohorts reporting intakes as low as 5–10 g/day, particularly among individuals adhering to restrictive renal diets. Such low intake levels reflect both reduced consumption of fruits, vegetables, and whole grains and concerns regarding potassium or phosphorus content in plant-based foods [47,48].

From a nutritional epidemiology perspective, low fiber intake is not merely a surrogate marker of poor diet quality, but a biologically relevant exposure that directly contributes to gut microbial alterations. Reduced availability of fermentable substrates limits saccharolytic fermentation in the colon and promotes a metabolic shift toward proteolytic pathways, particularly in the context of higher animal protein intake typical of Western dietary patterns [49].

#### 4.4.2. Gut Microbiota Alterations and Dysbiosis

Dietary fiber is a key determinant of gut microbiota composition and metabolic activity. Adequate fiber intake supports microbial diversity and favors the growth of saccharolytic bacteria capable of producing SCFAs, such as acetate, propionate, and butyrate. In CKD, reduced fiber intake contributes to dysbiosis characterized by decreased abundance of beneficial genera (e.g., *Bifidobacterium, Lactobacillus, Faecalibacterium*) and overrepresentation of proteolytic and toxin-producing taxa, including Proteobacteria and Enterobacteriaceae [49,50].

This dysbiotic profile is further exacerbated by uremia-related factors, including increased colonic urea diffusion, elevated luminal ammonia, altered intestinal transit, and frequent exposure to medications such as antibiotics, iron supplements, and phosphate binders. Collectively, these factors impair gut barrier integrity and increase intestinal permeability, facilitating translocation of endotoxins and microbial metabolites into the systemic circulation [49].

#### 4.4.3. Production of Gut-Derived Uremic Toxins

A central consequence of fiber deficiency and dysbiosis in CKD is the increased generation of gut-derived uremic toxins, particularly IS, PCS, and, to a lesser extent, TMAO. These compounds arise predominantly from bacterial fermentation of aromatic amino acids, tryptophan, tyrosine, and phenylalanine, processes that are amplified when fermentable carbohydrates are scarce and proteolytic fermentation predominates [49,50].

Experimental and clinical evidence indicates that IS and PCS exert direct nephrotoxic and vasculotoxic effects, promoting oxidative stress, endothelial dysfunction, tubular injury, and interstitial fibrosis, while also contributing to systemic inflammation and cardiovascular risk. Importantly, these protein-bound uremic toxins are poorly cleared by dialysis and tend to accumulate as kidney function declines [49].

Conversely, increasing dietary fiber intake—either through fiber-rich foods or targeted supplementation—shifts colonic metabolism toward saccharolytic fermentation, enhances SCFA production, and reduces circulating concentrations of IS and PCS. A review of randomized controlled trials (n = 21 trials, total n = 700 participants; intervention duration 4–16 weeks) found that fiber supplementation (6–50 g/day) significantly reduced circulating indoxyl sulfate (SMD −0.34, 95% CI −0.57 to −0.12) and p-cresyl sulfate (SMD −0.22, 95% CI −0.42 to −0.02), alongside reductions in inflammatory markers, including interleukin-6 (SMD −0.44, 95% CI −0.73 to −0.16) and tumor necrosis factor-α (SMD −0.34, 95% CI −0.66 to −0.02), supporting a clinically meaningful modulation of both gut-derived uremic toxins and systemic inflammation in individuals with chronic kidney disease [48].

Beyond toxin reduction, SCFAs derived from fiber fermentation play a protective role by strengthening gut barrier integrity, modulating immune responses, and exerting anti-inflammatory and epigenetic effects through G-protein-coupled receptors and histone deacetylase inhibition. The loss of these protective metabolites in low-fiber diets further amplifies the inflammatory and metabolic burden characteristic of CKD [49,50,51].

Taken together, the available evidence supports dietary fiber intake as a central modulator of the gut–kidney axis. Within the framework of dietary transitions, reduced fiber consumption emerges as a key mechanistic link between Western dietary patterns, gut dysbiosis, increased uremic toxin burden, and CKD progression. These findings reinforce the importance of plant-forward dietary strategies and fiber adequacy as integral components of CKD prevention and management, moving beyond traditional nutrient restriction toward optimization of dietary quality.

To improve clarity and synthesize the growing body of epidemiological evidence, Table 1 summarizes key cohort studies, randomized trials, and meta-analyses examining the relationship between dietary exposures, including ultra-processed foods, dietary acid load, sodium intake, and fiber intake and CKD risk or progression.

Table 1 provides a structured synthesis of the epidemiological and clinical evidence supporting the conceptual framework proposed in this review. By integrating findings from population-based cohort studies, randomized controlled trials, systematic reviews, and meta-analyses, the table summarizes the current state of evidence linking major dietary exposures to CKD outcomes. Specifically, it captures studies evaluating dietary acid load (e.g., NEAP, PRAL, or DAL indices), UPF consumption according to the NOVA classification, sodium intake and restriction interventions, overall dietary pattern quality (e.g., Mediterranean- or DASH-type diets), and dietary fiber supplementation.

This comparative synthesis highlights several important features of the current evidence base. First, there is consistency of evidence across diverse study designs and populations, with multiple cohort studies and pooled analyses reporting associations between acidogenic diets, UPF-rich dietary patterns, or high sodium exposure and increased CKD risk or adverse renal outcomes. Second, several studies demonstrate dose–response relationships, particularly for UPF consumption and dietary acid load indices, where higher exposure categories are associated with progressively greater risk of CKD incidence or markers of renal injury. Third, the evidence base reflects triangulation across complementary methodological approaches, combining long-term observational cohorts that capture population-level exposures, randomized controlled trials evaluating dietary interventions such as sodium restriction or fiber supplementation, and meta-analytic syntheses that integrate findings across heterogeneous settings.

Taken together, this triangulated evidence supports a coherent interpretation: contemporary dietary environments characterized by high intake of ultra-processed foods, elevated dietary acid load, and reduced fiber intake contribute to CKD risk and progression through multiple interconnected pathways. These pathways include sodium-driven hypertension, metabolic acidosis, gut–kidney axis dysregulation with increased uremic toxin generation, and systemic inflammatory and endocrine responses. By consolidating these findings into a single comparative framework, Table 1 functions as an evidence integration tool that enhances transparency, facilitates cross-study comparison, and strengthens the translational relevance of the emerging link between global dietary transitions and kidney health.

## 5. Metabolic and Inflammatory Complications

Dietary transitions contribute to CKD-related metabolic and inflammatory complications through a “double-hit” model: (i) promotion of obesity and insulin resistance, raising CKD incidence and worsening prognosis; and (ii) amplification of chronic low-grade inflammation, a hallmark of CKD that accelerates vascular damage, protein-energy wasting risk, and cardiovascular complications [22,32]. Ultra-processed dietary patterns are central here because they combine high energy density, low fiber, and pro-inflammatory nutrient/additive profiles in a way that is difficult to disentangle using single-nutrient approaches [22,30].

Obesity and insulin resistance as intermediates and accelerants. At the population level, the nutrition transition has been tightly linked to rising obesity and type 2 diabetes, which are dominant upstream drivers of CKD and major determinants of progression and cardiovascular events. Beyond classic “metabolic CKD,” higher adiposity itself is increasingly recognized as a contributor to CKD risk even in the absence of overt metabolic disease, plausibly via hyperfiltration, adipokine dysregulation, RAAS activation, and inflammation [32]. KDIGO’s 2025 conference report on obesity in CKD underscores the strength of the epidemiologic association across large meta-analytic datasets and emphasizes obesity as a modifiable but structurally driven exposure requiring both clinical and public health responses [34]. In this context, dietary environments rich in UPFs and low in minimally processed foods increase obesity risk through reduced satiety signaling, higher palatability-driven intake, and lower fiber/protein quality ultimately contributing to insulin resistance and dysglycemia.

### 5.1. Inflammation: A Mechanistic Bridge from UPFs to CKD Complications

Low-grade systemic inflammation is central to the CKD phenotype and links strongly to atherosclerosis, sarcopenia risk, anemia of chronic disease, and frailty. UPF-dominant diets have been repeatedly associated with inflammatory markers and adverse cardiometabolic outcomes in evidence syntheses, with proposed mechanisms including gut microbiome alterations, increased intestinal permeability, oxidative stress, and higher exposure to certain food additives and packaging-related chemicals [22,30,52]. Mechanistic reviews highlight gut dysbiosis and systemic inflammation as key pathways connecting UPFs to CVD and cancer; in CKD, these may interact with uremic toxin generation and impaired clearance, further intensifying inflammatory signaling [29,52]. Importantly, the association between UPFs and CKD outcomes appears to persist even after adjustment for conventional diet quality indices in recent meta-analytic work, suggesting that UPFs may capture risk-relevant features not fully reflected in nutrient-based scoring systems (e.g., additives, matrix effects, food structure) [53]. Importantly, the association between UPF consumption and CKD-related outcomes has been reported to remain statistically significant after multivariable adjustment for major cardiometabolic covariates, including age, sex, blood pressure, diabetes status, body mass index, and lifestyle factors.

### 5.2. Clinical Relevance: Metabolic-Inflammation Synergy in CKD

At the population level, the nutrition transition has been tightly linked to rising obesity and type 2 diabetes, which are dominant upstream drivers of CKD and major determinants of progression and cardiovascular events. Beyond classic “metabolic CKD,” higher adiposity itself is increasingly recognized as an independent contributor to CKD risk, even in the absence of overt metabolic disease, plausibly via hyperfiltration, adipokine dysregulation, activation of the renin–angiotensin–aldosterone system, and chronic low-grade inflammation. A recent large-scale meta-analysis of prospective cohorts reported that overweight and obesity were significantly associated with increased risk of incident CKD, with a dose–response relationship observed per 5 kg/m^2^ increment in BMI, supporting adiposity as a causal and graded determinant of renal risk. These findings reinforce obesity as a modifiable yet structurally driven exposure requiring both clinical and population-level responses [29]. This has direct implications for prevention: population measures that reduce UPF consumption may lower obesity and insulin resistance rates, while also reducing inflammation burden, thereby indirectly attenuating CKD complications.

## 6. Mineral and Bone Disorders

CKD-MBD is increasingly understood as a systemic syndrome involving bone, vasculature, endocrine pathways (PTH-vitamin D-FGF23 axis), and inflammation. Dietary transitions are relevant to CKD-MBD because ultra-processed diets often increase exposure to highly bioavailable inorganic phosphate additives (“hidden phosphorus”), while simultaneously displacing minimally processed foods with lower additive burden and different phosphorus bioavailability profiles [54,55,56]. This shift has implications not only for biochemical control in established CKD, but also for population-level cardiovascular risk given the links between phosphate/FGF23 dysregulation and vascular pathology [57].

The pathophysiological relevance of dietary transitions in CKD-MBD extends beyond total phosphorus intake to the chemical form and metabolic handling of phosphate exposure. Inorganic phosphate salts commonly used in ultra-processed foods such as sodium phosphate, calcium phosphate, and phosphoric acid are absorbed with markedly higher efficiency than organic phosphorus naturally present in plant or animal foods. This near-complete bioavailability results in acute phosphate loading, which stimulates FGF23 secretion from osteocytes and suppresses 1,25-dihydroxyvitamin D synthesis. These early endocrine adaptations alter the PTH–vitamin D-FGF23 axis and may precede overt hyperphosphatemia. Experimental and translational data suggest that sustained phosphate excess contributes to vascular smooth muscle cell osteogenic transformation, medial vascular calcification, and adverse cardiac remodeling. Thus, additive-driven phosphate exposure represents a biologically plausible mechanism linking ultra-processed dietary patterns to cardio–renal–bone dysregulation [54,55,56,57].

### Hidden Phosphorus from Additives: Why It Matters

Inorganic phosphate additives are widely used in processed and ultra-processed foods for texture, moisture retention, leavening, and shelf-life extension. Critically, additive phosphate is absorbed far more efficiently than organic phosphorus naturally present in foods, which makes additive-heavy diets particularly problematic for patients with CKD who rely on dietary phosphate restriction to mitigate hyperphosphatemia and secondary hyperparathyroidism. Common examples include processed meats (e.g., deli meats, sausages, and reconstituted poultry products) and cola beverages, where phosphate-containing additives are frequently used and may substantially increase total phosphorus exposure without being readily apparent to consumers. Calvo et al. outline how industrial phosphate additives may mechanistically contribute to cardiorenal disease via phosphate loading and endocrine responses (FGF23 and PTH), potentially promoting vascular calcification and cardiac remodeling [54,55]. Biruete et al. further emphasize the evolving complexity of dietary phosphorus management and the need to incorporate additive sources into practical guidance since conventional nutrient databases often underestimate phosphate additives and labeling may be incomplete or inconsistent [54].

An important clinical consideration is that phosphate retention and overt hyperphosphatemia typically occur in later stages of CKD, whereas elevations in fibroblast growth factor 23 (FGF23) can be detected earlier (often during CKD stages 2–3) as a compensatory endocrine response to maintain phosphate homeostasis. Most mechanistic and longitudinal data linking elevated FGF23 to adverse cardiovascular remodeling and event risk derive from CKD cohorts, especially in mild-to-moderate stages of renal impairment. Although associations between higher FGF23 levels and cardiovascular outcomes have also been observed in general population studies, the magnitude and clinical relevance of these associations appear stronger in individuals with impaired kidney function. These findings support the plausibility that additive phosphate exposure in ultra-processed food-dominant diets may contribute to CKD-CVD coupling through early endocrine dysregulation [56,57]. Complementing this, cardiovascular-focused reviews highlight phosphate dysregulation as an important contributor to vascular disease in impaired kidney function [57,58].

From a nutritional epidemiology and policy standpoint, “hidden phosphorus” is a structural exposure: it is driven by food manufacturing practices and availability, not only by individual preference. This aligns with the American Society of Nephrology Kidney Health Guidance (2025), which proposes a tiered approach to assessing and mitigating risk from potassium and phosphorus additives, underscoring gaps in labeling and calling for better transparency to protect kidney health [59]. At the population level, increasing reliance on packaged staples can shift phosphate exposure upward without corresponding awareness, particularly in LMICs undergoing rapid nutrition transitions. This suggests a role for labeling reform, additive disclosure, and reformulation as CKD-MBD prevention complements to clinical management.

## 7. Dietary Determinants of CKD Progression and Secondary Prevention

Dietary transitions influence progression through proteinuria control, BP regulation, acid–base balance, additive exposures, and overall dietary pattern quality [60]. Increasingly, the epidemiologic literature supports the clinical intuition that diet is not only relevant to CKD incidence but also to the tempo of decline, especially when examined through dietary patterns and high-impact exposures like sodium and UPFs.

### 7.1. Dietary Patterns and Accelerated Decline

Evidence syntheses indicate that healthier dietary patterns are associated with more favorable kidney outcomes, although the strength of evidence varies by endpoint. Most consistent benefits have been observed for intermediate markers, particularly reductions in proteinuria and slower decline in estimated eGFR. Umbrella-level reviews report that low-protein diets can reduce proteinuria and, in selected populations, very-low-protein diets may delay progression to ESKD. In the 2025 umbrella review including 28 meta-analyses, the largest pooled analysis (19 trials; 2492 participants) showed that low-protein diets reduced urinary protein excretion by −0.44 g/day (95% CI −0.80 to −0.08; *p* = 0.02), with even larger effects observed in diabetic kidney disease (SMD −2.26; 95% CI −2.99 to −1.52; *p* < 0.001) [23]. Regarding hard renal endpoints, the Cochrane-prioritized meta-analysis (17 trials; 2996 participants with CKD stages 3–5) reported that very-low-protein diets significantly reduced the risk of ESKD (RR 0.64; 95% CI 0.49–0.85; *p* = 0.002), whereas standard low-protein diets did not (RR 1.05; 95% CI 0.73–1.53; *p* = 0.78) [23]. Importantly, no significant effect was observed on all-cause mortality (LPD RR 0.77; 95% CI 0.51–1.18), underscoring that benefits appear primarily on proteinuria and renal progression markers rather than mortality. Evidence for hard renal endpoints such as dialysis initiation or kidney failure remains more limited and is largely derived from short- to medium-term trials and observational cohorts rather than long-term randomized studies. Plant-forward and DASH-like patterns have similarly been associated with improvements in albuminuria and eGFR trajectories, although heterogeneity in study design and adherence complicates direct comparisons [19]. More broadly, plant-based dietary patterns have been synthesized as associated with lower CKD incidence and slower CKD progression, with attention to the distinction between healthy and unhealthy plant-based patterns [19]. These findings align with a prevention framework in which improving dietary pattern quality (rather than single nutrient targets alone) offers scalable benefits.

### 7.2. Sodium Reduction and Proteinuria: A Core Secondary Prevention Target

Among single dietary components, sodium stands out because of its relatively rapid and reproducible effects on BP and proteinuria, two key predictors of CKD progression. Randomized trials of sodium restriction in CKD—typically short-term (2–12 weeks) have shown clinically meaningful reductions in blood pressure and, in some studies, albuminuria/proteinuria, although long-term data on eGFR slope and ESKD remain scarce. For example, in a prospective, open-label randomized controlled trial by Trakarnvanich et al. (2024) [27], involving 194 participants with CKD stages 1–3 followed for 3 months, a low-salt diet (1.5 g/day) resulted in a significantly greater reduction in systolic blood pressure compared with usual diet (−6.57 mmHg vs. −0.58 mmHg; between-group difference −5.99 mmHg, 95% CI −11.24 to −0.74; *p* = 0.025). Although albuminuria did not significantly change between groups, the decline in eGFR over 3 months was attenuated in the intervention group (−1.49 mL/min/1.73 m^2^) compared with controls (−3.01 mL/min/1.73 m^2^; *p* = 0.013 within-group for controls), supporting sodium restriction as a core secondary prevention strategy in early-stage CKD [27].

Nonetheless, systematic reviews have emphasized variability in evidence across outcomes and methodological challenges (measurement of sodium intake, adherence, and confounding), underscoring the need for longer-term pragmatic trials and food-environment interventions that make sodium reduction feasible [32]. The key public health insight is that sustained sodium reduction is difficult when UPFs dominate the food supply, linking progression prevention to structural dietary transition drivers.

### 7.3. Ultra-Processed Foods and Faster Decline: Emerging Epidemiologic Signal

Beyond sodium, UPFs may accelerate progression via multiple converging mechanisms: higher sodium and phosphate additive load, lower fiber and potassium density, pro-inflammatory properties, and associations with obesity/insulin resistance [53]. A recent systematic review and meta-analysis by Xiao et al. (2024) [39], including nine cohort studies, reported that higher UPF consumption was significantly associated with increased risk of incident CKD. The pooled estimates demonstrated a graded relationship across exposure categories, and subgroup analyses suggested consistency across geographic regions and study designs. These findings strengthen the epidemiologic evidence that UPF exposure may contribute not only to CKD incidence but also to more rapid decline in kidney function [39]. This emerging evidence strengthens the argument that dietary transitions toward UPF dominance may contribute to faster progression in susceptible groups, making UPF reduction a plausible secondary prevention target.

### 7.4. Clinical and Public Health Relevance for Secondary Prevention

Secondary prevention requires integrating evidence into feasible strategies: sodium reduction, UPF displacement, and pattern-based counseling that is adaptable to CKD stage and comorbidity. KDIGO’s 2024 CKD guideline emphasizes individualized dietary adaptation (including sodium, phosphorus, potassium, and protein) and the importance of trained renal nutrition providers, an approach consistent with applying dietary pattern evidence while managing CKD-specific risks [61,62]. At the population level, this supports a two-level model: (i) clinical nutrition therapy for people with CKD, and (ii) upstream food policy and reformulation to reduce sodium density and additive exposures, thereby enabling adherence and reducing progression risk.

**Figure 2:**
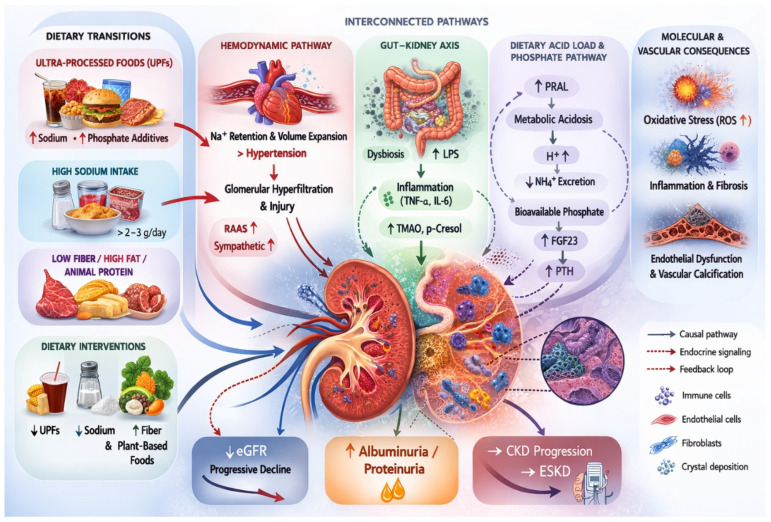
Conceptual framework illustrating the interconnected pathways linking dietary transitions to chronic kidney disease.

The Figure 2 summarizes how shifts toward ultra-processed, sodium-dense, low-fiber, and animal-protein-dominant dietary patterns contribute to CKD development and progression through three major interconnected biological axes: (1) the hemodynamic pathway (sodium retention, volume expansion, RAAS activation, hypertension, and glomerular hyperfiltration); (2) the gut–kidney axis (dysbiosis, endotoxemia, uremic toxin production, systemic inflammation); and (3) the dietary acid load and phosphate pathway (metabolic acidosis, reduced ammonium excretion, increased FGF23 and PTH signaling). These processes converge on shared molecular mechanisms—including oxidative stress, endothelial dysfunction, inflammation, and fibrosis—ultimately leading to albuminuria, progressive eGFR decline, and CKD progression to ESKD. The framework integrates epidemiologic, mechanistic, and clinical evidence to illustrate how dietary environments may act as upstream determinants of cardio-kidney-metabolic risk.

## 8. Conclusions

CKD has emerged as a major yet underrecognized non-communicable disease whose growing global burden closely parallels contemporary dietary transitions. This narrative review integrates evidence from nutritional epidemiology, clinical research, and population health studies to show that shifts toward ultra-processed, sodium-dense, and low-fiber dietary patterns are largely associated across most cohorts with increased CKD risk, complications, and progression through interconnected cardiometabolic, inflammatory, and gut-mediated pathways.

Taken together, this evidence supports a reframing of CKD as a condition shaped not only by individual clinical factors but also by broader food environments and structural dietary exposures, particularly in low- and middle-income countries undergoing rapid nutrition transitions. From a nutritional epidemiology perspective, improving overall diet quality and reducing reliance on ultra-processed foods emerge as coherent, biologically plausible targets for CKD prevention across the life course.

Integrating dietary pattern-based evidence into clinical nephrology, public health strategies, and food policy may offer scalable opportunities to attenuate CKD burden. From a clinical perspective, three practical priorities emerge: (i) sustained reduction of dietary sodium to improve blood pressure control and limit proteinuria; (ii) progressive displacement of ultra-processed foods with minimally processed, plant-forward alternatives to address cardiometabolic and inflammatory pathways; and (iii) careful attention to hidden sources of highly bioavailable phosphate additives, particularly in processed meats and cola beverages, to mitigate endocrine and vascular complications. Addressing these upstream dietary determinants, alongside individualized medical management, is likely essential to achieve meaningful and equitable reductions in CKD-related morbidity and mortality.

## Figures and Tables

**Table 1:** Summary key epidemiological and clinical studies linking dietary exposures with chronic kidney disease risk and progression.

Study (Year)	Design/Setting	Population (n; Age)	Exposure (Definition)	Outcome (s)	Main Effect Estimate (s)	Key Covariates/Focus
Ko et al. 2017 [42]	Observational cohort, Korea	n = 1369 elderly adults	Dietary acid load (NEAP from protein and potassium intake)	CKD prevalence	Highest vs. lowest NEAP quartile: OR: 2.30 (95% CI 1.16–4.60)	Age, sex, BMI, lifestyle, comorbidities
Wang et al. 2024 [44]	Cross-sectional analysis (China Health and Nutrition Survey)	n = 7749 adults	Dietary acid load (NEAP and PRAL indices)	CKD prevalence	Higher NEAP associated with higher CKD risk (multivariable models)	Sociodemographics, diet, metabolic factors
Huang et al. 2025 [45]	Hospital-based observational study, China	n = 300 CKD patients with T2DM	Dietary acid–base load (DAL)	CKD severity/metabolic parameters	DAL associated with CKD risk in women. OR: 6.47 (95% CI 1.19–35.18)	Age, BMI, energy intake, eGFR
Du et al. 2022 [40]	Prospective cohort (ARIC)	n = 14,679 adults	Ultra-processed food intake (NOVA classification)	Incident CKD	Highest vs. lowest UPF consumption associated with =24% higher CKD risk	Demographics, lifestyle, cardiometabolic risk
Xiao et al. 2024 [39]	Systematic review and meta-analysis of cohort studies	4 cohort studies (219,132 participants)	Ultra-processed food consumption	Incident CKD	Pooled estimate RR = 1.25 (95% CI 1.18–1.33) (highest vs. lowest intake)	Multi-cohort adjustment
He et al. 2021 [34]	Systematic review and meta-analysis	17 observational studies (149,958 participants)	Dietary patterns (Mediterranean, DASH, plant-based)	CKD incidence	Healthy dietary patterns associated with lower CKD risk	Diet quality indices
Li et al. 2025 [23]	Umbrella review of meta-analyses	28 meta-analyses	Dietary patterns and protein intake	Proteinuria, CKD progression	Low-protein diets reduced proteinuria; very-low-protein diets RR = 0.64 (95% CI 0.49–0.85) for ESKD	Synthesized evidence
Trakarnvanich et al. 2024 [27]	Randomized controlled trial	n = 194 CKD stage 1–3	Low-salt diet (1.5 g/day sodium)	BP, eGFR decline	SBP reduction −6.57 and DBP −6.95 mmHg vs. control	Randomized trial
Wathanavasin et al. 2025 [48]	Systematic review and meta-analysis of RCTs	21 RCTs; n = 700 CKD patients	Fiber supplementation (=6–50 g/day)	Uremic toxins, inflammation	Indoxyl sulfate SMD −0.34, p-cresyl sulfate SMD −0.22, Blood urea nitrogen SMD −0.25	Experimental trials
Kanbay et al. 2025 [31]	14 Clinical trials review	General and CKD populations	Ultra-processed foods	Cardio-kidney-metabolic outcomes	UPFs linked to cardiometabolic and renal risk	Mechanistic pathways

**Abbreviations:** CKD = chronic kidney disease UPF = ultra-processed foods DAL = dietary acid load NEAP = net endogenous acid production PRAL = potential renal acid load RCT = randomized controlled trial RR = relative risk OR = odds ratio SMD = standardized mean difference. **Notes:** Effect estimates correspond to the most fully adjusted models reported in each study or pooled estimates from systematic reviews.

## Data Availability

No new data were created or analyzed in this study. Data sharing is not applicable to this article.

## Abbreviations

The following abbreviations are used in this manuscript:

ARIC	Atherosclerosis Risk in Communities Study
BP	Blood pressure
CKD	Chronic kidney disease
CKD–MBD	Chronic kidney disease-mineral and bone disorder
CKM	Cardio-kidney-metabolic
CVD	Cardiovascular disease
DAL	Dietary acid load
DASH	Dietary Approaches to Stop Hypertension
eGFR	Estimated glomerular filtration rate
ESKD	End-stage kidney disease
FFQs	Food Frequency Questionnaires
FGF23	Fibroblast growth factor 23
GBD	Global Burden of Disease
HEI	Healthy Eating Index
IS	Indoxyl sulfate
KDIGO	Kidney Disease: Improving Global Outcomes
LMICs	Low- and middle-income countries
MeSH	Medical Subject Headings
Na/K	Sodium-to-potassium ratio
NCDs	Non-communicable diseases
NEAP	Net endogenous acid production
NOVA	Food classification system based on degree of processing
PCS	p-cresyl sulfate
PRAL	Potential renal acid load
PTH	Parathyroid hormone
RAAS	Renin–angiotensin–aldosterone system
RCT	Randomized controlled trial
RR	Relative risk
SCFAs	Short-chain fatty acids
SMD	Standardized Mean Difference
TMAO	Trimethylamine N-oxide
UPFs	Ultra-processed foods

## References

[1] GBD 2023 Kidney Failure with Replacement Therapy Collaborators (2025). Global, regional, and national prevalence of kidney failure with replacement therapy and associated aetiologies, 1990–2023: A systematic analysis for the Global Burden of Disease Study 2023. Lancet Glob. Health.

[2] GBD Chronic Kidney Disease Collaboration (2020). Global, regional, and national burden of chronic kidney disease, 1990–2017: A systematic analysis for the Global Burden of Disease Study 2017. Lancet.

[3] Jha V., Garcia-Garcia G., Iseki K., Li Z., Naicker S., Plattner B., Saran R., Wang A.Y., Yang C.W. (2013). Chronic kidney disease: Global dimension and perspectives. Lancet.

[4] Kovesdy C.P. (2022). Epidemiology of chronic kidney disease: An update 2022. Kidney Int. Suppl..

[5] Matsushita K., van der Velde M., Astor B.C., Woodward M., Levey A.S., de Jong P.E., Coresh J., Gansevoort R.T., Chronic Kidney Disease Prognosis Consortium (2010). Association of estimated glomerular filtration rate and albuminuria with all-cause and cardiovascular mortality. Lancet.

[6] Stanifer J.W., Muiru A., Jafar T.H., Patel U.D. (2016). Chronic kidney disease in low- and middle-income countries. Nephrol. Dial. Transplant..

[7] Popkin B.M. (2001). The nutrition transition and obesity in the developing world. J. Nutr..

[8] Popkin B.M., Adair L.S., Ng S.W. (2012). Global nutrition transition and the pandemic of obesity in developing countries. Nutr. Rev..

[9] Monteiro C.A., Cannon G., Levy R.B., Moubarac J.C., Louzada M.L., Rauber F., Khandpur N., Cediel G., Neri D., Martinez-Steele E. (2019). Ultra-processed foods: What they are and how to identify them. Public Health Nutr..

[10] Mozaffarian D. (2016). Dietary and policy priorities for cardiovascular disease, diabetes, and obesity. Circulation.

[11] Monteiro C.A., Moubarac J.C., Levy R.B., Canella D.S., Louzada M.L.D.C., Cannon G. (2018). Household availability of ultra-processed foods and obesity in nineteen European countries. Public Health Nutr..

[12] Baker P., Machado P., Santos T., Sievert K., Backholer K., Hadjikakou M., Russell C., Huse O., Bell C., Scrinis G. (2020). Ultra-processed foods and the nutrition transition: Global, regional and national trends, food systems transformations and political economy drivers. Obes. Rev..

[13] Calvo M.S., Uribarri J. (2013). Public health impact of dietary phosphorus excess on bone and cardiovascular health. Am. J. Clin. Nutr..

[14] Juul F., Vaidean G., Lin Y., Deierlein A.L., Parekh N. (2021). Ultra-processed foods and incident chronic kidney disease. J. Am. Coll. Cardiol..

[15] Khatri M., Moon Y.P., Scarmeas N., Gu Y., Gardener H., Cheung K., Wright C.B., Sacco R.L., Nickolas T.L., Elkind M.S. (2014). The association between a Mediterranean-style diet and kidney function in the Northern Manhattan Study. Clin. J. Am. Soc. Nephrol..

[16] Rebholz C.M., Crews D.C., Grams M.E., Steffen L.M., Levey A.S., Miller E.R., Appel L.J., Coresh J. (2016). DASH (Dietary Approaches to Stop Hypertension) Diet and Risk of Subsequent Kidney Disease. Am. J. Kidney Dis..

[17] Kelly J.T., Palmer S.C., Wai S.N., Ruospo M., Carrero J.J., Campbell K.L., Strippoli G.F.M. (2017). Healthy Dietary Patterns and Risk of Mortality and ESRD in CKD: A Meta-Analysis of Cohort Studies. Clin. J. Am. Soc. Nephrol..

[18] Lin J., Hu F.B., Curhan G.C. (2011). Association of dietary patterns with albuminuria and kidney function decline in older white women: A subgroup analysis from the Nurses’ Health Study. Am. J. Kidney Dis..

[19] Kim H., Caulfield L.E., Garcia-Larsen V., Steffen L.M., Grams M.E., Coresh J., Rebholz C.M. (2019). Plant-based diets and incident CKD. Clin. J. Am. Soc. Nephrol..

[20] Cole N.I., Swift P.A., He F.J., MacGregor G.A., Suckling R.J. (2019). The effect of dietary salt on blood pressure in individuals receiving chronic dialysis: A systematic review and meta-analysis of randomised controlled trials. J. Hum. Hypertens..

[21] Mozaffarian D., Angell S.Y., Lang T., Rivera J.A. (2018). Role of government policy in nutrition-Barriers to and opportunities for healthier eating. BMJ.

[22] Lane M.M., Gamage E., Du S., Ashtree D.N., McGuinness A.J., Gauci S., Baker P., Lawrence M., Rebholz C.M., Srour B. (2024). Ultra-processed food exposure and adverse health outcomes: Umbrella review of epidemiological meta-analyses. BMJ.

[23] Li J., Yang L., Yang B., Liu C., Wei W., Huang Y., Ren J., Wang B., Ma L., Zhang L. (2025). Dietary Patterns and Kidney Health: An Umbrella Review of Systematic Reviews and Meta-Analyses. Nutr. Rev..

[24] Amir S., Kim H., Hu E.A., Ricardo A.C., Mills K.T., He J., Fischer M.J., Pradhan N., Tan T.C., Navaneethan S.D. (2024). Adherence to Plant-Based Diets and Risk of CKD Progression and All-Cause Mortality: Findings From the Chronic Renal Insufficiency Cohort (CRIC) Study. Am. J. Kidney Dis..

[25] Díaz-Haaz D.I., Espinoza-Pérez E.A., Aguilar-Alonso J.A., Megchún-Hernández M., Gonzalez-Diaz F., García-Chong N.R. (2025). Effects of fibroblast growth factor 23 (FGF23) on the Cardiovascular System: A Review of Literature. Cureus.

[26] Verma A., Popa C. (2023). The Interplay Between Dietary Sodium Intake and Proteinuria in CKD. Kidney Int. Rep..

[27] Trakarnvanich T., Chailimpamontree W., Kantachuvesiri S., Anutrakulchai S., Manomaipiboon B., Ngamvitchukorn T., Suraamornkul S., Trakarnvanich T., Kurathong S. (2024). Effect of a Low Salt Diet on the Progression of Chronic Kidney Disease: A Prospective, Open-Label, Randomized Controlled Trial. J. Prim. Care Community Health.

[28] Zwager C.L., Esseghir M.I., van Vliet A.M.C., Daams J.G., Vogt L., Olde Engberink R.H.G. (2025). Estimated Dietary Na^+^/K^+^-Ratio and Cardiovascular Disease: A Systematic Review and Meta-Analysis. Kidney Blood Press. Res..

[29] Dai S., Wellens J., Yang N., Li D., Wang J., Wang L., Yuan S., He Y., Song P., Munger R. (2024). Ultra-processed foods and human health: An umbrella review and updated meta-analyses of observational evidence. Clin. Nutr..

[30] Lichtenstein A.H., Appel L.J., Vadiveloo M., Hu F.B., Kris-Etherton P.M., Rebholz C.M., Sacks F.M., Thorndike A.N., Van Horn L., Wylie-Rosett J. (2021). 2021 Dietary Guidance to Improve Cardiovascular Health: A Scientific Statement From the American Heart Association. Circulation.

[31] Kanbay M., Ozbek L., Guldan M., Abdel-Rahman S.M., Narin A.E., Ortiz A. (2025). Ultra-processed foods and cardio-kidney-metabolic syndrome: A review of recent evidence. Ultra-processed foods and cardio-kidney-metabolic disease: Integrated review. Eur. J. Intern. Med..

[32] Avesani C.M., Cuppari L., Nerbass F.B., Lindholm B., Stenvinkel P. (2023). Ultraprocessed foods and chronic kidney disease-double trouble. Clin. Kidney J..

[33] Kramer H. (2019). Diet and chronic kidney disease. Adv Nutr..

[34] He L.Q., Wu X.H., Huang Y.Q., Zhang X.Y., Shu L. (2021). Dietary patterns and chronic kidney disease risk: A systematic review and updated meta-analysis of observational studies. Nutr. J..

[35] Sullivan V.K., Rebholz C.M. (2023). Nutritional Epidemiology and Dietary Assessment for Patients With Kidney Disease: A Primer. Am. J. Kidney Dis..

[36] Avesani C.M., Cecchini V., Sabatino A., Lindholm B., Stenvinkel P., Canella D.S., Picard K. (2025). Ultra-processed foods and food additives in chronic kidney disease: Unveiling hidden risks and advocating smarter food choices. Clin. J. Am. Soc. Nephrol..

[37] Lou L.M., Vercet A., Caverní A., Medrano C., Lou E., Munguía P., Sanz A. (2021). Impact of ultra-processed food consumption on chronic kidney disease. Nefrologia.

[38] Cecchini V., Sabatino A., Contzen B., Avesani C.M. (2026). Food additives containing potassium, phosphorus, and sodium in ultra-processed foods: Potential harms to individuals with chronic kidney disease. Eur. J. Clin. Nutr..

[39] Xiao B., Huang J., Chen L., Lin Y., Luo J., Chen H., Fu L., Tang F., Ouyang W., Wu Y. (2024). Ultra-processed food consumption and the risk of incident chronic kidney disease: A systematic review and meta-analysis of cohort studies. Ren. Fail..

[40] Du S., Kim H., Crews D.C., White K., Rebholz C.M. (2022). Association between ultra-processed food consumption and risk of incident chronic kidney disease: A prospective cohort study. Am. J. Kidney Dis..

[41] He X., Zhang X., Si C., Feng Y., Zhu Q., Li S., Shu L. (2024). Ultra-processed food consumption and chronic kidney disease risk: A systematic review and dose–response meta-analysis. Front. Nutr..

[42] Ko B.J., Chang Y., Ryu S., Kim E.M., Lee M.Y., Hyun Y.Y., Lee K.B. (2017). Dietary acid load and chronic kidney disease in elderly adults: Protein and potassium intake. PLoS ONE.

[43] Hamidianshirazi M., Ekramzadeh M. (2021). Dietary acid load and chronic kidney disease. Saudi J. Kidney Dis. Transplant..

[44] Wang S., Fan X., Zheng X., Xia P., Zou H., Zhang Z., Chen L. (2024). Association between dietary acid load and chronic kidney disease in the Chinese population: A comprehensive analysis of the China Health and Nutrition Survey (2009). Nutrients.

[45] Huang H., Wang Q., Zhang R., Liu F., Niu Y., Luo Y., Dong Z. (2025). Correlation between dietary acid–base load and chronic kidney disease patients with type 2 diabetes mellitus. Front. Nutr..

[46] Zarantonello D., Brunori G. (2023). The role of plant-based diets in preventing and mitigating chronic kidney disease: More light than shadows. J. Clin. Med..

[47] Camerotto C., Cupisti A., D’Alessandro C., Muzio F., Gallieni M. (2019). Dietary fiber and gut microbiota in renal diets. Nutrients.

[48] Wathanavasin W., Cheungpasitporn W., Thongprayoon C., Fülöp T. (2025). Effects of dietary fiber supplementation on modulating uremic toxins and inflammation in chronic kidney disease patients: A systematic review and meta-analysis of randomized controlled trials. Toxins.

[49] Cigarrán S., González E., Cases A. (2017). Intestinal microbiota in chronic kidney disease. Nefrologia.

[50] Alobaidi S. (2025). The gut–kidney axis in chronic kidney disease: Mechanisms, microbial metabolites, and microbiome-targeted therapeutics. Front. Med..

[51] Cigarrán S. (2023). Is the contribution of fiber with prebiotics justified in chronic kidney disease? Influence on uremic toxins: Utility or fiction?. Nutr. Hosp..

[52] Furth S.L., Colhoun H.M., Kanbay M., Kukla A., Lim L.L., MacLaughlin H.L., Navaneethan S.D., Shroff R., Stenvinkel P., Cheung M. (2025). The relationship between obesity and chronic kidney disease: Conclusions from a Kidney Disease: Improving Global Outcomes (KDIGO) Controversies Conference. Kidney Int..

[53] Liu C., Young A., Abi N., Chen J., Fernandes Gyorfy M., Li Y., Sun S., Zhou J.J., Sun Y.V. (2025). Novel approaches and applications in identifying DNA methylation markers of cardio-kidney-metabolic disease. Epigenomics.

[54] Leonberg K.E., Maski M.R., Scott T.M., Naumova E.N. (2025). Ultra-Processed Food and Chronic Kidney Disease Risk: A Systematic Review, Meta-Analysis, and Recommendations. Nutrients.

[55] Biruete A., Hill Gallant K.M., Lloyd L., Meade A., Moe S.M., St-Jules D.E., Kistler B.M. (2023). ‘Phos’tering a Clear Message: The Evolution of Dietary Phosphorus Management in Chronic Kidney Disease. J. Ren. Nutr..

[56] Calvo M.S., Dunford E.K., Uribarri J. (2023). Industrial Use of Phosphate Food Additives: A Mechanism Linking Ultra-Processed Food Intake to Cardiorenal Disease Risk?. Nutrients.

[57] Raju S., Saxena R. (2025). Hyperphosphatemia in Kidney Failure: Pathophysiology, Challenges, and Critical Role of Phosphorus Management. Nutrients.

[58] Turner M.E., Beck L., Hill Gallant K.M., Chen Y., Moe O.W., Kuro-O M., Moe S.M., Aikawa E. (2024). Phosphate in Cardiovascular Disease: From New Insights Into Molecular Mechanisms to Clinical Implications. Arter. Thromb. Vasc. Biol..

[59] Biruete A., Anderson C., Bernier-Jean A., Clase C.M., Clegg D., Crews D.C., Denburg M., Hill Gallant K.M., Gutierrez O.M., Ix J.H. (2025). ASN Kidney Health Guidance Workgroup on Food Additives. ASN Kidney Health Guidance on Potassium and Phosphorus Food Additives. J. Am. Soc. Nephrol..

[60] Ketteler M., Evenepoel P., Holden R.M., Isakova T., Jørgensen H.S., Komaba H., Nickolas T.L., Sinha S., Vervloet M.G., Cheung M. (2025). Chronic kidney disease-mineral and bone disorder: Conclusions from a Kidney Disease: Improving Global Outcomes (KDIGO) Controversies Conference. Kidney Int..

[61] Shi H., Su X., Li C., Guo W., Wang L. (2022). Effect of a low-salt diet on chronic kidney disease outcomes: A systematic review and meta-analysis. BMJ Open.

[62] Cheung A.K., Chang T.I., Cushman W.C., Furth S.L., Hou F.F., Ix J.H., Knoll G.A., Muntner P., Pecoits-Filho R., Sarnak M.J. (2021). Executive summary of the KDIGO 2021 Clinical Practice Guideline for the Management of Blood Pressure in Chronic Kidney Disease. Kidney Int..

